# Comment on van wegen et al.: the association between urgency level and hospital admission, mortality and resource utilization in three emergency department triage systems

**DOI:** 10.1186/s13049-025-01414-2

**Published:** 2025-05-26

**Authors:** Amir Mirhaghi

**Affiliations:** PhD in Nursing, Assistant Professor of Nursing, Nursing and Midwifery Care Research Center, Mashhad University of Medical Sciences, Mashhad, Iran

Dear Editor,

The manuscript titled “*The association between urgency level and hospital admission*,* mortality and resource utilization in three emergency department triage systems: an observational multicenter study*” by van Wegen et al., was reviewed with great interest. This study compared the performance of the Manchester Triage System (MTS), the Emergency Severity Index (ESI), and the Netherlands Triage Standard (NTS) by investigating the association between urgency levels and resource utilization, hospitalization and in-hospital mortality in Emergency Department (ED) patients [1]. They reported that there was a remarkably high percentage of in-hospital mortality in the least urgent category for the NTS (12.4%), when compared with the MTS (6.3%) and the ESI (0.8%). Therefore they suggested that the ESI may be more effective in distinguishing between patients with low and high urgency, with a reduced risk of undertriage when compared to the MTS and NTS. An important point that warrants emphasis is that the superiority of the ESI, compared to other triage systems, should not be interpreted as complete efficacy of scale. Further clarification about what subgroups of patients benefit the most from the scale is needed too.

While the validity and reliability of the ESI have been confirmed in literature [2], Mistriage rate of ESI remains noteworthy. For example, the study by Sax et al. reported a total mistriage rate of 32.2%, consisting of 28.9% overtriage and 3.3% undertriage [3]. Mistriage rate for early identification of patients who eventually died in the ED was 30% including 19% overtriage and 11% undertriage [1], consistent with previous findings. However, the 11% undertriage rate should not be overlooked, as it exceeds acceptable levels. But the point is, what does this mistriage mean clinically?

The accuracy of triage systems such as the Manchester Triage System and ESI in identifying dying patients is approximately 0.80, which is considered “good” but still falls short of the “excellent” threshold (> 0.90) for critically-ill patients and this is the best we have. Besides, it is crucial to note that these triage errors were observed across a broad range of patient complaints, and it is plausible that some patients` subgroups—such as those presenting with dyspnea or chest pain—are at even higher risk of triage inaccuracies. For instance, Vatankhah et al. reported a mistriage rate of 66% (65.5% overtriage and 1.3% undertriage) for patients with cardiopulmonary complaints in ESI group [4], a result that closely mirrors van Wegen findings. The mistriage rate for patients requiring ICU or CCU admission was 57%, comprising 17% overtriage and 40% undertriage in ESI group [1]. These figures highlight the challenge of accurately triaging patients with acute complaints, as determining the severity of their condition and assigning an appropriate triage level is often compromised. In contrast, patients with non-critical conditions—such as mild abdominal pain or minor limb injuries—can usually be categorized more easily. Consequently, the majority of mistriage involve patients with acute and critical complaints, with chest pain being the most significant. This is primarily due to the limited diagnostic evaluation for chest pain complaint, which heavily relies on ECG and troponin tests. Therefore, incorporating additional diagnostic tools—such as rapid troponin kits, electrocardiography, capnography, peak flow meters, and pulse oximetry—could significantly reduce triage errors and enhance diagnostic evaluation [4].

In the absence of diagnostic aids in triage room, triage staff often take a cautious approach, assigning higher acuity levels to minimize undertriage and improve sensitivity, though this leads to increased overtriage and reduced specificity. Furthermore, when examining the accuracy of these triage scales in distinguishing patients who require hospital admission from those who can be discharged, the accuracy rate falls below 0.6, which is considered “very poor” or almost at a chance (Table 1). This limitation is especially problematic in the triage of stable patients, as overcrowding in EDs is a common issue. Accurate triage of stable patients can play a key role in improving care for the critically ill patients. Another promising solution is to strengthen pre-hospital triage systems. Introducing a pre-hospital triage layer before hospital arrival may improve in-hospital triage accuracy and better direct patients to appropriate facilities. In van Wegen study, only 56% of critically ill patients (ESI levels 1 and 2) accessed ambulance services, whereas 26% of patients with stable conditions also used ambulances—possibly contributing to overtriage [1]. Enhancing telephone triage protocols at dispatch centers and improving the training and knowledge of pre-hospital emergency personnel could significantly improve triage outcomes and decision-making.

**Table 1:** Comparison of diagnostic evaluation criteria for Van Wegen study

Outcomes	Deceased at ED			Deceased in hospital			ICU/CCU Admission			Admission			Discharged		
Triage Scale	MTS	ESI	NTS	MTS	ESI	NTS	MTS	ESI	NTS	MTS	ESI	NTS	MTS	ESI	NTS
Sensitivity	0.94	0.89	0.96	0.60	0.58	0.55	0.67	0.60	0.72	0.74	0.95	0.62	0.38	0.18	0.40
Specificity	0.79	0.81	0.62	0.80	0.82	0.62	0.80	0.83	0.64	0.38	0.18	0.40	0.74	0.95	0.62
Positive predictive value	0.01	0.01	0.01	0.05	0.05	0.02	0.10	0.12	0.05	0.86	0.45	0.67	0.21	0.85	0.35
Negative predictive value	0.99	0.99	0.99	0.99	0.99	0.98	0.98	0.98	0.98	0.21	0.85	0.35	0.86	0.45	0.69
Accuracy	0.80	0.82	0.63	0.80	0.82	0.62	0.80	0.82	0.64	0.68	0.50	0.55	0.67	0.50	0.55
Undertriage %	6	11	4	40	42	45	33	40	38	26	5	38	62	82	60
Overtriage %	21	19	38	20	18	38	20	17	36	62	82	60	26	5	38
Mistriage %	27	30	42	60	60	83	53	57	74	88	87	98	88	87	98
Diagnostic table criteria*	Level 1 & 2 vs. Level 3 & 4									Level 1, 2 & 3 vs. Level 4			Level 4 vs. Level 1, 2 & 3		

*Data extracted from the Table 4 in the original manuscript. The 2 × 2 table includes a test variable (e.g. most & very urgent level (1&2) vs. urgent & non-urgent level (3&4)) and a classification variable (e.g. deceased vs. alive)

## Data Availability

No datasets were generated or analysed during the current study.
